# Metagenome-Assembled Genome Sequence of *Pseudomonas stutzeri* Strain CO183 Isolated from a Coalbed Methane Well

**DOI:** 10.1128/genomeA.01237-16

**Published:** 2016-11-03

**Authors:** Daniel E. Ross, Djuna Gulliver

**Affiliations:** a National Energy Technology Laboratory, Pittsburgh, Pennsylvania, USA; b AECOM, Pittsburgh, Pennsylvania, USA

## Abstract

A near-complete *Pseudomonas stutzeri* draft genome was extracted from a coalbed metagenome. The draft genome described herein provides insight into the functional pathways encoded by this bacterium and its potential role in coalbed methane environments.

## GENOME ANNOUNCEMENT

The ever-increasing demand for affordable domestic energy supplies necessitates alternative methods to maximize utilization of these supplies. One alternative method, microbial-enhanced coalbed methane, harnesses resident microbial populations for *in situ* natural gas production from unmineable coal seams (1). The *Pseudomonas stutzeri* strain CO183 draft genome was extracted from the assembled metagenome of coalbed methane cuttings. The sub-bituminous coal samples were retrieved from the Basal Coal Zone at a depth of 730 m. DNA extraction was performed by An and coworkers, as described previously (2). Illumina paired-end read data used in this study was obtained from the Hydrocarbon Metagenome Project database (http://hmp.ucalgary.ca).

Metagenome reads were assembled with metaSPAdes (3), binned with VizBin (4), and the most complete genome bin was assigned to the genus *Pseudomonas*. Specifically, metagenome contigs were mapped to multiple *P. stutzeri* strains (5), and a total of 866 contigs (4,740,859 bp) were mapped to *P. stutzeri* RCH2 (E-value cutoff = 1e^−20^). Using 833 *Pseudomonas*-specific marker genes in CheckM (6), the resultant genome bin was 92.84% complete and had 13.64% contamination. The mapped *Pseudomonas*-assigned contigs and the raw metagenome reads were used as input for paired-read iterative contig extension (PRICE) (7), and after 30 cycles the genome bin improved significantly, resulting in a bin of 117 contigs (4,522,918 bp) at 99.27% completeness and 0.4% contamination. The longest contig was 280,110 bp, the *N*_50_ was 74,098, and the GC content was 62.97%. The final draft genome bin had an average nucleotide identity of 97.59% with *P. stutzeri* RCH2.

The genome was annotated with Rapid Annotations using Subsystem Technology (RAST) version 2.0 using the RASTtk pipeline (8, 9) and the NCBI Prokaryotic Genomes Automatic Annotation Pipeline (PGAP) (http://www.ncbi.nlm.nih.gov/genome/annotation_prok). RAST estimated 4,396 coding sequences and 64 RNAs. PGAP estimated 4,429 total genes (4,066 coding genes), 68 RNA genes (four rRNAs, 60 tRNAs, and four ncRNAs), and 115 pseudogenes. Initial analysis suggests that the genome encodes for a complete tricarboxylic acid cycle, heavy-metal resistance (copper, chromium, cadmium, zinc, arsenic), and denitrification pathways. Future comparative analyses of *Pseudomonas stutzeri* strain CO183 with other *Pseudomonas* spp. found in coalbed methane environments will provide insight into the ecology of these strains, how they evolved, and their contribution to biogenic coal degradation.

### Accession number(s).

This whole-genome shotgun project has been deposited at DDBJ/ENA/GenBank under the accession number MCNJ00000000. The version described in this paper is the first version, MCNJ01000000.
